# Targeting the chemokine receptor CXCR4 with histamine analog to reduce inflammation in juvenile arthritis

**DOI:** 10.3389/fimmu.2023.1178172

**Published:** 2023-09-26

**Authors:** Nassima Bekaddour, Nikaïa Smith, Benoit Beitz, Alba Llibre, Tom Dott, Anne Baudry, Anne-Sophie Korganow, Sébastien Nisole, Richard Mouy, Sylvain Breton, Brigitte Bader-Meunier, Darragh Duffy, Benjamin Terrier, Benoit Schneider, Pierre Quartier, Mathieu P. Rodero, Jean-Philippe Herbeuval

**Affiliations:** ^1^ Centre National de la Recherche Scientifique (CNRS) Unité Mixte de Recherche (UMR)-8601, Université Paris Cité, Paris, France; ^2^ Chemistry and Biology, Modeling and Immunology for Therapy (CBMIT), Paris, France; ^3^ Translational Immunology Unit, Institut Pasteur, Université Paris Cité, Paris, France; ^4^ BIOASTER, Lyon, France; ^5^ Institut National de la Santé et de la Recherche Médicale (INSERM) Unité Mixte de Recherche (UMR)-S1124, Team Stem Cells, Signaling and Prions, Université Paris Cité, Paris, France; ^6^ Institut National de la Santé et de la Recherche Médicale (INSERM) Unité Mixte de Recherche (UMR) - S1109, Faculté de Médecine, Fédération Hospitalo-Universitaire OMICARE, Fédération de Médecine Translationnelle de Strasbourg (FMTS), Université de Strasbourg, Strasbourg, France; ^7^ Department of Clinical Immunology and Internal Medicine, National Reference Center for Rare Autoimmune Diseases Centre National de Référence des maladies auto-immunes et systémiques rares de Strasbourg (RESO), Hôpitaux Universitaires de Strasbourg, Strasbourg, France; ^8^ Unité de Formation et de Recherche (UFR) Medicine, University of Strasbourg, Strasbourg, France; ^9^ Institut de Recherche en Infectiologie de Montpellier (IRIM), Université de Montpellier, Centre National de la Recherche Scientifique (CNRS) Unité Mixte de Recherche (UMR) 9004, Montpellier, France; ^10^ Paediatric Haematology-Immunology and Rheumatology Department, Centre de référence des rhumatismes inflammatoires et maladies auto-immunes systémiques rares de l'enfant (RAISE) Reference Centre for Rare Diseases, Hôpital Universitaire Necker, Assistance Publique-Hôpitaux de Paris, Paris, France; ^11^ Paediatric Radiology Department, Necker-Enfants Malades University Hospital, Paris, France; ^12^ Pediatric Immunology-Hematology and Rheumatology Unit, Laboratory of Immunogenetics of Pediatric Autoimmune Diseases, INSERM U1163, Necker-Enfants Malades Hospital, Assistance Publique - Hôpitaux de Paris (APHP), Imagine Institute, Université Paris Cité, Paris, France; ^13^ Department of Internal Medicine, National Referral Center for Rare Systemic Autoimmune Diseases, Assistance Publique Hôpitaux de Paris-Centre (APHP-CUP), Université de Paris, Paris, France

**Keywords:** monocytes, cytokines, arthritis, inflammation, treatment

## Abstract

**Introduction:**

Among immune cells, activated monocytes play a detrimental role in chronic and viral-induced inflammatory pathologies, particularly in Juvenile Idiopathic Arthritis (JIA), a childhood rheumatoid arthritis (RA) disease. The uncontrolled activation of monocytes and excessive production of inflammatory factors contribute to the damage of bone-cartilage joints. Despite the moderate beneficial effect of current therapies and clinical trials, there is still a need for alternative strategies targeting monocytes to treat RA.

**Methods:**

To explore such an alternative strategy, we investigated the effects of targeting the CXCR4 receptor using the histamine analog clobenpropit (CB). Monocytes were isolated from the blood and synovial fluids of JIA patients to assess CB's impact on their production of key inflammatory cytokines. Additionally, we administered daily intraperitoneal CB treatment to arthritic mice to evaluate its effects on circulating inflammatory cytokine levels, immune cell infiltrates, joints erosion, and bone resorption, as indicators of disease progression.

**Results:**

Our findings demonstrated that CXCR4 targeting with CB significantly inhibited the spontaneous and induced-production of key inflammatory cytokines by monocytes isolated from JIA patients. Furthermore, CB treatment in a mouse model of collagen-induce arthritis resulted in a significant decrease in circulating inflammatory cytokine levels, immune cell infiltrates, joints erosion, and bone resorption, leading to a reduction in disease progression.

**Discussion:**

In conclusion, targeting CXCR4 with the small amino compound CB shows promise as a therapeutic option for chronic and viral-induced inflammatory diseases, including RA. CB effectively regulated inflammatory cytokine production of monocytes, presenting a potential targeted approach with potential advantages over current therapies. These results warrant further research and clinical trials to explore the full therapeutic potential of targeting CXCR4 with CB-like molecules in the management of various inflammatory diseases.

## Introduction

Rheumatoid arthritis (RA) is a long inflammatory condition that results to the aggressive synovial hyperplasia causing destruction of articular joints. Joint inflammation is characterized by proliferation of macrophage-like (1) and fibroblast-like synoviocytes to form a pannus, which invades and destroys the cartilage (2). Juvenile Idiopathic Arthritis (JIA) is a rare and complex form of RA affects children younger than 16 years old, characterized by a multifactorial disorder with heterogeneous manifestations that include all forms of chronic arthritis (3, 4). Over-production of TNF-α, IL-1β and IL-6 is strongly involved in most forms of JIA (5, 6). The release of such inflammatory factors by monocytes depends on several highly conserved families of pattern recognition receptors (PRR), one of them being the Toll-like receptor family (TLRs). The increase in circulating TNF-α levels in all forms of JIA argues for a major contribution of monocytes to disease progression (7). This is supported by the observation that monocytes for systemic JIA patients produced more inflammatory cytokines than monocytes from healthy donors in response to TLR4 and TLR8 stimulation (8). Current antirheumatic drugs, including corticosteroids and antibodies-based biotherapies, target inflammatory macrophages or macrophages secreted cytokines to reduce synovial inflammation. However, not all patients respond to antibody therapy and chronic application of glucocorticoids leads to severe side effects, highlighting the need for novel therapeutic strategies.

In the context of chronic and acute inflammatory diseases, the chemokine receptor CXCR4 could emerge a potential therapeutic target. We previously showed that histamine and the histamine analog clobenpropit (CB) through their engagement of CXCR4 exhibited a broad-spectrum inhibitory activity on the production of all subtypes of interferons (IFN) in TLR7-activated human plasmacytoid dendritic cells (pDCs) (9). Moreover, intranasal spray of CB resulted in drastic reduction of type I and III IFN secretions in broncho-alveolar lavages from Influenza A virus (IAV) infected mice (9). Of note, the anti-IFN activity of CB was not associated with histamine receptors but strictly dependent on CXCR4 (9). As CXCR4 is highly expressed by all immune cells, including monocytes and macrophages, we hypothesize that CB could also downmodulate monocyte-driven inflammation in rheumatoid arthritis.

In this study, we explored the potential of CB to decrease the production of proinflammatory cytokines by monocytes obtained from the blood and synovial fluids of individuals diagnosed with Juvenile Idiopathic Arthritis (JIA). In these monocytes, we evaluated both the spontaneous and induced cytokine production. Finally, we further probed *in vivo* whether CB treatment would attenuate inflammation and impair disease progression in a model of collagen-induced arthritic mice.

## Results

### CB down-regulates TNF-α, IL-1β, and IL-6 productions in TLR-7/8-activated monocytes

**Figure 1:**
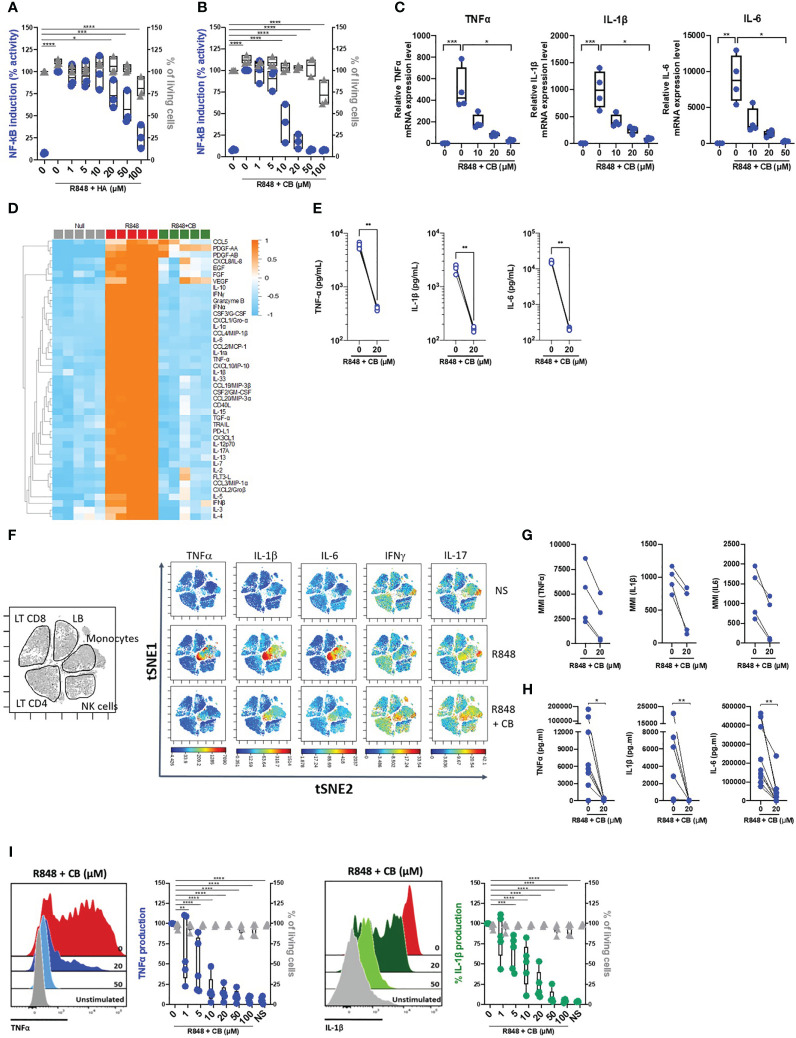
CB inhibited TLR-7/8-mediated TNF-α, IL-1β and IL-6 production by human monocytes. **(A, B)** THP1 NF-κB reporter cells were treated with increasing concentration of Histamine **(A)** or CB **(B)** ranging from 1 to 100 µM then stimulated with R848 (5 μg/ml) for 24 hours. NF-κB reporter activity was measured using QUANTI-Blue, a SEAP detection reagent. **(C)** mRNA levels of TNF-α, IL-1β and IL-6 from THP1-dual pre-incubated with increased doses of CB and stimulated overnight with R848 (5 μg/ml), were measured by RT-qPCR and normalized to RPL13A. Kruskal-Wallis with Dunn’s multiple comparisons test. **(D-G)** PBMCs from healthy donors (HD) were preincubated with CB (20 μM) then stimulated with R848 (5 μg/mL) overnight. **(D, E)** Cytokine production was measured in the supernatant using a bead-based multiplexed immunoassay system Luminex. **(D)** Heatmap representation of statistically different cytokines (P<0.05) between the different conditions (Null, R848, R848+CB), ordered by hierarchical clustering. Up-regulated cytokines are shown in orange, and down-regulated in blue. P values were determined with the Kruskal-Wallis test. **(E)** Individual cytokines from the Luminex are represented. Mann-Whitney test. **(F)** PBMCs were analyzed by mass cytometry (CyTOF) and tSNE analysis were performed using CD56, CD3, CD11c, BDCA4, CD14, HLADR, CD123, CD4, CD8, and CD19 markers. Intracellular levels of TNF-α, IL-1β, IL-6, IFNγ, and IL-17were evaluated. **(G)** Mean Metal Intensity (MMI) of TNF-α, IL-1β, and IL-6 was evaluated on gated monocytes from four different donors. **(H)** Cytokine production was measured in the supernatants of purified monocytes from nine HD using the multiplex bead-based immunoassay LEGENDplex. Mann-Whitney test. **(I)** Purified monocytes from five HD were preincubated with increased doses of CB then stimulated with R848 during 5 h. Intracellular levels of TNF-α and IL-1β as well as viability were evaluated by flow cytometry. SSC-A, side scatter. FSC-A, forward scatter. 2-way ANOVA. All data are presented as median ± range. ****P < 0.0001, ***P < 0.001, **P < 0.01, *P < 0.05.

To first assess the potential anti-inflammatory properties of histamine and the histamine analog CB, we took advantage of the monocyte-derived THP-1 NF-κB reporter cell line. Upon TLR7/8 activation by R848, the reporter gene SEAP is induced by activation of the NF-κB signaling pathway. We thus measured the production of SEAP by R848-activated THP-1 cells in the presence or absence of varying concentration of histamine (**Figure 1A**) or CB (**Figure 1B**) ranging from 1 to 100 µM. We showed that CB, and to a lesser extent histamine, reduced in a dose-dependent manner activation of NF-κB in THP-1 cells, without any obvious toxicity. In addition, CB treatment (20 µM) prevented the transcription of TNF-α, IL-1β and IL-6 encoding genes in R848-activated THP-1 cells (**Figure 1C**).

To extend our results obtained with THP-1 cell line, we next tested the ability of CB to control cytokine production in R848-stimulated human blood mononuclear cells (PBMCs) from healthy donors (HD). We first tested the overall effect of CB by measuring the concentrations of multiple cytokines (*i.e.* IL-1β, -6, -8, -10, TNF- α …), chemokines (*i.e.* CCL2, CCL3, CCL4, CCL5…), growth factors (*i.e.* EGF, FGF, VEGF…) and interferons (IFNα/β/γ) in the cell culture medium of R848-stimulated PBMCs with or without of CB (20 µM) (n=5) (**Figure 1D**). CB downmodulated the R848-induced production of chemokines, growth factors, IFN subtypes, and all proinflammatory cytokines, including TNF-α, IL-1β and IL-6 (**Figures 1D, E**). Mass Cytometry (CyTOF) analysis revealed that among PBMCs monocytes represented the main producers of TNF-α, IL-1β and IL-6 upon TLR7/8 stimulation by R848. CB treatment drastically reduced the production of those inflammatory factors by R848-activated monocytes (**Figures 1F, G**) (n=4). To better evaluated the effect of CB on the proinflammatory cytokines, we purified monocytes from the blood of HD. Accordingly, using a multiplex bead-based assay (**Figure 1H**) and intracellular flow cytometry staining of TNF-α and IL-1β, we showed in monocytes purified from HD that CB inhibited the production of TNF-α and IL-1β (IC_50 = _2,8 μM and IC_50 = _8 μM, respectively) with no observed toxicity (**Figure 1I**).

### CB controls activation of monocytes from healthy individuals in a CXCR4-dependent manner

**Figure 2:**
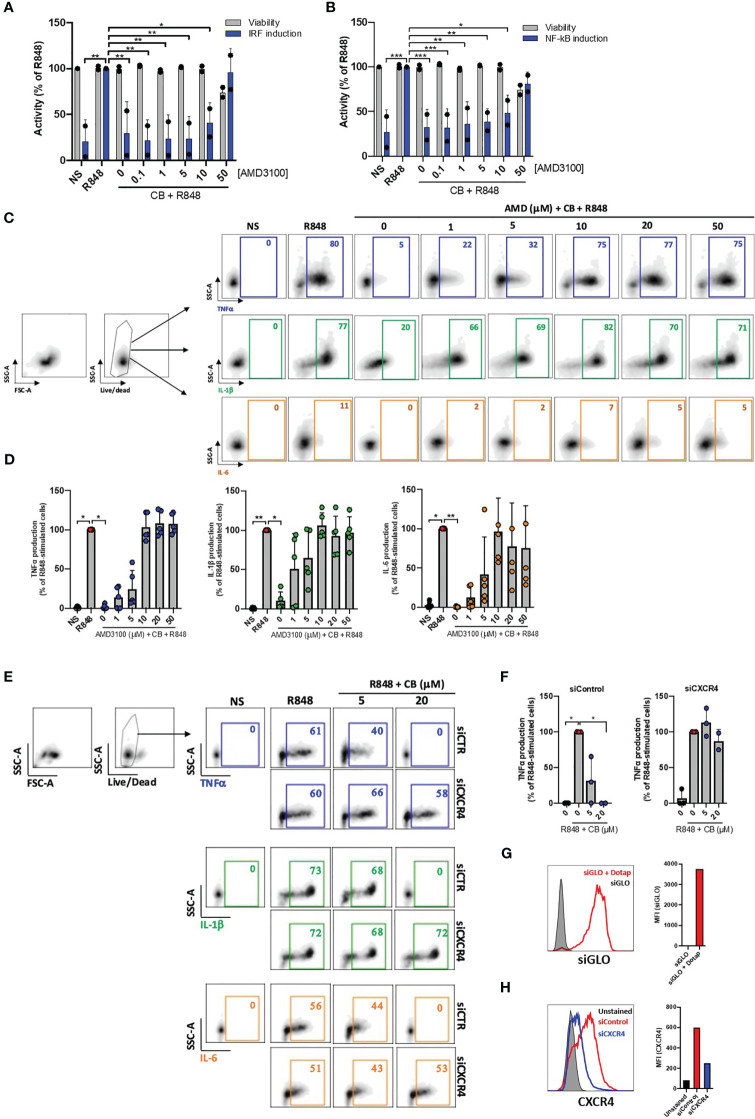
CB immunoregulatory activity on primary monocytes is CXCR4 dependent. **(A)** and **(B)** PBMCs from two healthy donors were cultured in presence of increased concentrations of AMD3100, then treated with CB (20 µM) and activated with R848 (5 µg/mL) for 24 hours. The level of **(A)** interferons and **(B)** inflammatory cytokines in the culture supernatants was measured using the reporter cell line THP1-dual. Two-way ANOVA with Dunnett’s multiple comparisons test. **(C)** and **(D)** Isolated monocytes from five healthy donors were cultured in presence of increased concentrations of AMD3100, then treated with CB (20 µM) and activated with R848 (1 µg/mL) for 6 hours. Intracellular levels of TNF-α, IL-1β and IL-6 were evaluated by flow cytometry, **(C)** dot plot representation from a donor and **(D)** histogram representation from 5 donors. Friedman with Dunn’s multiple comparisons test. **(E, F)** Monocytes were treated for 24 hours with CXCR4 siRNA (siCXCR4) or Control siRNA (siControl) at 160 nM. Cells were then pre-incubated with CB (20 μM) and stimulated with R848 (1µg/mL) for 6 hours. Intracellular levels of TNF-α, IL-1β and IL-6 were evaluated by flow cytometry: **(E)** dot plot representation from a donor and **(F)** histogram representation from 3 donors. Data shown as the median +/-s.d. Krustal-Wallis with Dunn’s multiple comparisons test. **(G)** the efficiency of transfection with dotap on monocytes was evaluated with GLO siRNA in flow cytometry. **(H)** The expression of CXCR4 on monocytes treated with siControl and siCXCR4 was assessed by flow cytometry. **P < 0.01, *P < 0.05. NS, nonstimulated.

We next tested whether the anti-inflammatory properties of CB depend on CXCR4. In our initial approach, we employed the widely recognized CXCR4 antagonist AMD3100 to regulate the anti-inflammatory effects of CB on activated PBMCs. Cells were cultured with increased concentrations of AMD3100 in presence of CB (20μM) and R848 for 24h. The supernatants were transferred to reporter THP1-Dual cells allowing simultaneous quantification of the activity of IRF and NF-κB promoters. We showed that CB reduced activation of both IRF and NF-κB and that AMD3100 blocked the anti-inflammatory activity of CB in a concentration dependent manner (**Figures 2A, B**).

We further quantified TNF-α, IL-1β and IL-6 productions in R848-stimulated primary purified monocytes from HD in the presence of CB (20 µM) and increasing concentrations of AMD3100 (up to 50 µM) using flow cytometry. As expected, AMD3100 inhibited in a dose-dependent manner the ability of CB to reduce intracellular TNF-α, IL-1β and IL-6 levels in monocytes without noticeable toxicity (**Figures 2C, D**).

To firmly establish that CB immunoregulatory activity relates to CXCR4, the expression of CXCR4 was silenced in primary monocytes using small interfering RNA (siRNA). While the transfection of a siRNA control (siControl) had no impact on the CB-induced inhibition of pro-inflammatory cytokines, CB lost its ability to inhibit TNF-α, IL-1β and IL-6 intracellular productions in CXCR4-silenced human monocytes (siCXCR4) (**Figures 2E, F**). Taking into account the 95% transfection efficiency in monocytes, we checked that CXCR4 expression was strongly reduced in siCXCR4-transfected monocytes compared to siControl (**Figures 2G, H**). These overall data show that the immunoregulatory effects of CB strictly depends on CXCR4 engagement.

### CB inhibits spontaneous proinflammatory cytokine production in JIA patients’ monocytes

**Figure 3:**
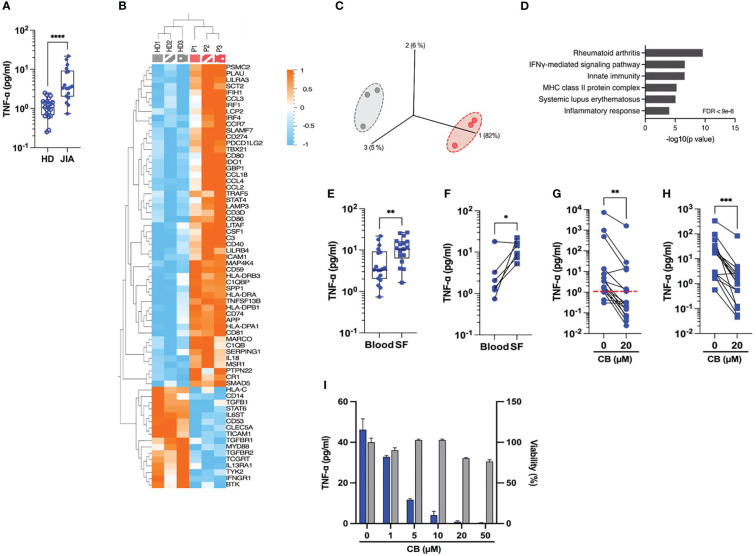
CB controls spontaneous inflammation in cells from JIA patients. **(A)** TNF-α was measured in the plasma of blood from HD and JIA patients by digital ELISA (Simoa). Box and whisker plots with median ± range. Mann-Whitney test. **(B)** Monocytes from blood from three healthy donors (HD) and three oligoJIA patients were isolated and total RNA extracted from the cells was isolated and subsequently analyzed using the NanoString nCounter system. Heat map illustrates the fold increase in mRNA expression levels of genes that exhibited significant differential expression between the two groups. (P < 0.05, fold change > 1.5). **(C)** PCA was performed based on genes from the array differentially expressed between the HD (gray) and the JIA patients (red). (P < 0.05, fold change > 1.5). **(D)** David pathway analysis was used for pathway enrichment analysis of differentially upregulated genes in JIA patients compared to the HD. **(E)** TNF-α was dosed in the plasma from blood or synovial fluids (SF) of JIA patients by Simoa. Mann-Whitney test. **(F)** TNF-α levels in paired plasma and SF from six different donors were dosed by Simoa. Wilcoxon test. TNF-α was measured in the supernatant of monocytes by digital ELISA (Simoa) purified from PBMCs of twenty JIA patients **(G)** or SFMCs of seventeen JIA patients **(H)**. Average baseline of HD is represented by the red dashed line. Mann-Whitney test. **(I)** Monocytes from one JIA patient were treated with various concentration of CB for 16h and TNF-α was measured in the supernatant by digital ELISA (Simoa). ****P < 0.0001, ***P < 0.001, **P < 0.01, *P < 0.05.

Our next attempt was to prob whether CB exerts an anti-inflammatory effect on immune cells isolated from JIA patients. Spontaneous inflammation is a well-known hallmark of JIA. While elevated levels of IL-6 are easily measurable in the plasma of RA patients (10), the detection of circulating TNF-α in these patients remains a challenge. Thus, to capture TNF-α in the plasma, we developed a TNF-α digital ELISA (Simoa) that detects TNF-α for concentrations as low as 1 fg/mL. Using this technology, we measured detectable levels of TNF-α in patients’ plasma and found higher concentration of TNF-α in blood plasma from JIA patients than in HD (JIA patients’ median = 3.4 pg/mL *vs.* HD’ median = 1.07 pg/mL) (**Figure 3A**). To better capture the spontaneous inflammation in JIA patients, we conducted a gene expression analysis on a set of 579 markers associated with inflammation on purified monocytes of PBMC from HD and JAI patients. As compared to HD, the expression of 51 inflammatory genes significantly increased and 16 decreased in oligoJIA patients (**Figure 3B**). The results of the principal components analysis (PCA) revealed that monocytes in each group grouped together (**Figure 3C**). Pathway analysis using DAVID (11, 12) showed enrichment in the rheumatoid arthritis pathway (**Figure 3D**). Since JIA is primarily a joint-related inflammatory disease (13), this prompted us to examine the inflammatory status of synovial fluids. Using Simoa technology, we measured TNF-α concentrations between 3 and 26 pg/mL (median = 10.8 pg/mL) in synovial fluid (SF) from eighteen JIA patients’ knees with active arthritis (**Figure 3E**). When comparing circulating levels of TNF-α in plasma and SF from six matched patients, higher levels of TNF-α were systematically measured in SF compared to blood plasma (**Figure 3F**).

We next assessed the effect of CB treatment on the spontaneous TNF-α production by human monocytes isolated from PBMCs (**Figure 3G**) and synovial fluid mononuclear cells (SFMCs) (**Figure 3H**) from JIA patients. CB treatment significantly decreased TNF-α levels in the culture medium of both SFMCs and PBMCs monocytes. We also showed that CB exerted a dose-dependent anti-inflammatory effect in purified monocytes from SF of one patient without any significant toxicity (**Figure 3I**).

Altogether, these data provide evidence that CB inhibits the spontaneous production of inflammatory cytokines by blood and synovial human monocytes within an arthritic context.

### CB attenuates R848-induced cytokine production in JIA patients’ cells

**Figure 4:**
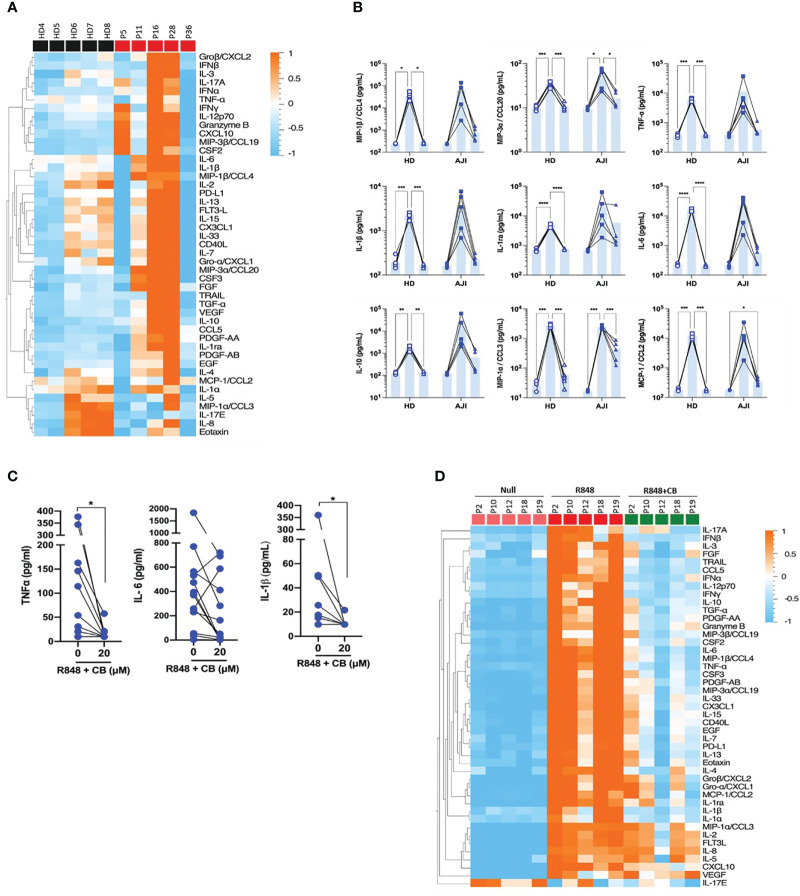
CB controls R848-induced inflammation in cells from JIA patients. **(A)** PBMCs from HD and JIA patients were incubated with R848 (5 µg/mL) for 16h. P2 P12 P18 and P19 patients are oligoJIA, P10 is an polyJIA. Cytokine production was measured in the supernatant using a bead-based multiplexed immunoassay system Luminex. Heatmap representation of statistically different cytokines (P<0.05) in PBMCs supernatant upon R848 stimulation between HD and JIA patients, ordered by hierarchical clustering. Mann-Whitney test. **(B)** PBMCs from HD and JIA patients were pre-incubated with CB at 20 µM and then stimulated with R848 (5 µg/mL) for 16h. Cytokine production was measured in the supernatant using a bead-based multiplexed immunoassay system Luminex. Individual cytokines are represented. Two-way RM ANOVA with Tukey *post hoc* correction. **(C)** Monocytes from JIA patients were pre-incubated with CB at 20 µM and then stimulated with R848 (5 µg/mL) for 16h. IL-6, IL-1β and TNF-α production was measured in the supernatant using the multiplex bead-based immunoassay LEGENDplex. Mann-Whitney test. **(D)** SFMCs were pre-incubated with CB at 20 µM and then stimulated with R848 (5 µg/mL) for 16h. P5 P11 and P28 patients are oligoJIA, P16 is a polyJIA and P36 an sJIA. Cytokine production was measured in the supernatant using a bead-based multiplexed immunoassay system Luminex. ****P < 0.0001, ***P < 0.001, **P < 0.01, *P < 0.05.

We next evaluated whether CB would also inhibit cytokine production by stimulated immune cells from JIA patients. To this purpose, we first compared cytokine production by PBMCs from HD and JIA patients using the 45-plex bead-based immunoassay Luminex. We showed that PBMCs from three JIA patients out (P11, P16 and P28) of five secreted more cytokines than those of HD (**Figure 4A**). This result confirms that cells from JIA patients are hyperresponsive to TLR-7/8 stimulation (8). Furthermore, CB reduced R848-induced inflammatory cytokine production by HD PBMCs as well as inflammatory cytokine hypersecretion by cells from JIA patients, and most drastically CCL20, CCL2 and CCL3 (**Figure 4B**). As monocytes are the main producers of proinflammatory cytokines, we purified monocytes from blood patients’ suffering from oligo articular (n=20), polyarticular (n=8) or systemic (n=5) JIA, or enthesitis-related arthritis (ERA) (n=3). For all subtypes of JIA, CB treatment drastically reduced levels of IL-1β and TNF-α and reduced level of IL-6 in the culture medium of R848-activated patients’ monocytes (**Figure 4C**).

Finally, using purified SFMCs from JIA patients we showed that CB inhibited the production of the 40 soluble factors induced by R848 activation (**Figure 4D**). Overall data indicate that CB could reduce hyper secretion of inflammatory cytokines observed in JIA patients during flares.

### CB inhibits inflammatory cytokine and reduces disease progression in collagen-induced arthritis mouse model

The anti-inflammatory properties of CB in PBMCs, SFMCs and purified monocytes of JIA patients, suggest that CB represents a promising therapeutic option for arthritis. Consequently, we proceeded to evaluate the potential therapeutic impact of CB in DBA/1J mice, which serve as a mouse model for arthritis-like pathology known as collagen-induced arthritis (CIA) (14). CIA mice exhibit numerous clinical, histological, and immunological similarities to human RA (15). This includes symmetric joint involvement, synovitis, cartilage, and bone erosions. Several studies reported a major role of IL-1β in disease development in CIA mouse model (16), as well as a pathologic role of IL-6 in the effector phase of autoimmune arthritis by promoting bone destruction (17).

**Figure 5:**
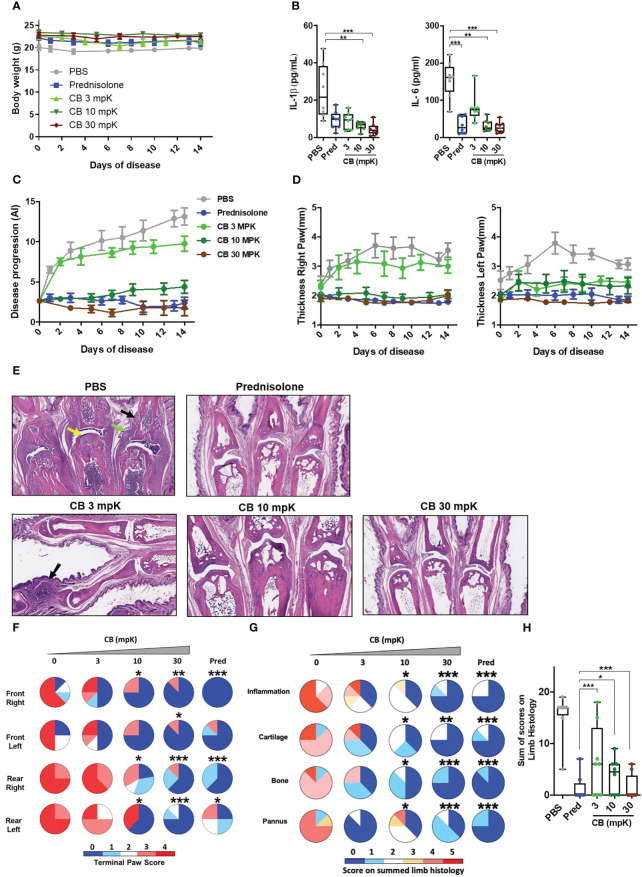
CB controls inflammation and disease onset in collagen induced arthritic mice. **(A)** The body weight of collagen induced arthritic mice treated with prednisolone (Pred, 15 mpK) or 3, 10, or 30 mpK of CB was measured every other day. **(B)** IL-1β and IL-6 were measured by ELISA in the serum after 2 weeks of treatment. Kruskal-Wallis test with Dunn’s *post hoc* correction. **(C)** the progression of the disease was assessed according to several markers in the legs. **(D)** The thickness of the legs was measured. **(E)** Representative hematoxylin and eosin staining of paw sections from collagen induced arthritic mice treated with prednisolone (Pred, 15 mpK) or 3, 10, or 30 mpK of CB: joints with pannus and inflammation (green arrow), loss of articular cartilage (yellow arrow) and bone remodeling (black arrow). **(F)** Impact of the treatment was evaluated by a paw score, signs of arthritis in all paws according to a 0-4 scale of ascending severity, after 2 weeks of treatments. **(G)** Effects of the treatment on the scoring for inflammation, cartilage damage, bone remodeling, and pannus after 2 weeks of treatment. **(H)** Combined disease score after 2 weeks of treatment. Kruskal-Wallis test with Dunn’s *post hoc* correction. All data are presented as median ± range, ***P < 0.001, **P < 0.01, *P < 0.05. *n* = 8 mice per group.

CIA mice were daily intraperitoneally injected with CB (at 3 mg/kg (mpK), 10 mpK, and 30 mpK) over a two-week period. As a reference drug, the corticosteroid prednisolone (15 mpK) was also injected in CIA mice. CB treatment did not exert any effect on mouse body weight whatever the dose tested overtime (**Figure 5A**), indicating no major toxicity of CB in treated mice. While CIA mice displayed elevated levels of circulating IL-1β and IL-6 two weeks after disease onset (**Figure 5B**), CB treatment resulted in a profound decrease of both cytokines (**Figure 5B**). Of note, the highest dose of CB (30 mpK) was as efficient as the referenced corticosteroid prednisolone.

Additionally, we conducted an investigation to assess the impact of CB treatment on the progression of the disease.The daily injection of CB attenuated the progression of the disease during the 14 days of treatment (**Figure 5C**). Mice treated with CB had thinner paws than mice treated with PBS (**Figure 5D**). In arthritic mice, the histological analysis of the tissue injury revealed severe cartilage damage (yellow arrow), bone remodeling (black arrow) and tissue infiltration by immune cells (green arrow) (**Figure 5E**). Daily CB treatment attenuated all these pathological markers **(** **Figure 5E**). To further characterize the effect of CB treatment, individual paws were scored, reflecting the severity of the disease. CB treatment drastically reduced the terminal paw score (**Figure 5F**), which was associated with reduced inflammation, cartilage damage, bone remodeling on limbs and pannus (**Figures 5G, H**).

## Discussion

In this study, we reveal a broad-spectrum anti-inflammatory activity of the histamine analog CB. Our findings reveal a notable decrease in the production of key inflammatory cytokines when monocytes derived from the blood and synovial fluids of individuals with JIA are exposed to CB. This reduction is observed both in spontaneous and induced cytokine production. This immuno-modulatory activity of CB strictly depends on the engagement of CXCR4 chemokine receptor. In addition, CB displays high efficiency *in vivo* to reduce inflammation and subsequent tissue damage in arthritic mice, leading to reduction of disease progression. In summary, our results strongly indicate that the targeting of CXCR4 using CB-like molecules holds great potential as a therapeutic approach for inflammatory diseases characterized by detrimental hyperactivation of monocytes.

The pro-inflammatory cytokines TNF-α and IL-6 are major contributors to JIA and RA (18, 19). The clinical benefits observed upon inhibiting TNF-α and IL-6 strongly support the hierarchical significance of these two pro-inflammatory factors among all the factors produced during JIA flares. While the TNF-α signature has been clearly identified in JIA patients (20), systemic TNF-α protein remains, however, difficult to detect and measure, notably because most classical ELISAs are not sensitive enough to detect TNF-α concentration below one pg/mL. To overcome this challenge, we developed an ultrasensitive digital TNF-α ELISA (Simoa) with attomolar sensitivity that permits to show that TNF-α concentration in plasma from JIA patients is six-fold higher than the one in healthy donors. Through Simoa experiments, we also measured that TNF-α concentration in synovial fluid JIA patients is higher in their synovial fluid than that in plasma. In most forms of RA, including JIA, blood monocytes are attracted to synovial fluids where they differentiate into inflammatory macrophages (1) and produce joint-degrading mediators. These immune cells emerge as being the major sources of TNF-α. Accordingly, we show that purified monocytes from JIA patients spontaneously produce more TNF-α than monocytes from healthy donors. As previously described by Cepika et al. (1), we also confirm that monocytes from JIA patients are hyperresponsive to TLR-7/8 activation compared to healthy individuals. Our study provides evidence that CB is a powerful inhibitor of pro-inflammatory cytokine and chemokine production in inflammatory monocytes derived from the blood or synovial fluid of individuals with Juvenile Idiopathic Arthritis (JIA). Interestingly, CB anti-inflammatory activity is not restricted to TNF-α and IL-6, as the inflammatory signature of JIA patients is largely toned down under CB exposure. These *ex vivo* results suggest that CB potentially reduces hypersecretion of cytokines and chemokines observed in JIA patients during flares. In RA animal models, IL-6 has been shown to promote osteoclast activation, synoviocyte proliferation, and recruitment to inflammatory areas, leading to the development of synovial pannus (17). In conjunction with IL-1β, IL-6 enhances the production of matrix metalloproteinases, thereby contributing to the degradation of joints and cartilage (21). In collagen-induced arthritis (CIA) mice, daily treatment of CB exhibits strong anti-inflammatory properties by blocking both IL-1β and IL-6 secretions. According to massive reduction of cartilage damage, bone remodeling, immune cell tissue infiltration and pannus CB treatment results in reduced disease progression and paw thickness in arthritic mice similarly to the referenced corticosteroid prednisolone. These *in vivo* experiments validate the concept of the immunomodulatory activity of CB observed *in vitro* and *ex vivo* on synovial monocytes from JIA patients.

In competition assays using the CXCR4 antagonist AMD3100 or siRNA-based experiments to down-regulate CXCR4 expression, we further demonstrated that CB anti-inflammatory activity on monocytes strictly depends on CXCR4. Beyond the impact of CXCR4 signaling on regulation of the IFN pathway (9), this result highlights that CXCR4 also represents a broad spectrum regulator of inflammation in various cell types, including pDC and monocytes. The concurrent anti-IFN and anti-inflammatory effects exerted by CXCR4 can have substantial clinical advantages, particularly considering the frequent presence and functional activity of type I interferons (IFNs) in RA (22). It is worth noting that a recent study has revealed significantly elevated levels of CXCR4 and its natural ligand, CXCL12, in both the serum and joint synovial fluids of individuals with active RA when compared to a control group (23). Furthermore, the expression levels of CXCR4 and CXCL12 in the active RA group were found to be higher compared to the group in remission (23). Higher accessibility to CXCR4 in RA patients makes targeting the CXCR4 anti-inflammatory pathway a particularly promising strategy for these pathologies.

For instance, drugs that target TNF-α, IL-6 and IL-1β cytokines or their receptors have shown beneficial effects in JIA patients (18). However, these treatments are often associated with highly heterogeneous responses across patients in terms of efficacy and treatment resistance. Consequently, it is not uncommon for a patient to change medication throughout the course of the disease. To some extent, this could be explained by the very high specificity of these treatments toward a single cytokine in a set of diseases characterized by a very broad inflammatory spectrum. By contrast to targeted therapies, corticosteroids have been used for decades to block overall pro-inflammation in RA patients, with, however, strong side-effects (24). In order to address these limitations, innovative approaches such as Janus kinase (JAK) inhibitors have emerged as promising strategies. These small molecules effectively inhibit the activity of JAK, and they have demonstrated reasonable success in treating the adult form of arthritis (25–27). Regarding strategies targeting JAK, our findings demonstrate that CB treatment effectively inhibits a broad range of cytokines *in vitro*, ex vivo, and *in vivo*.

In contrast, CB exerts its effects at an earlier stage compared to JAK inhibitors. Instead of directly targeting cytokine-mediated signaling, CB acts one step upstream by inhibiting the production of inflammatory cytokines. This approach may offer certain advantages in terms of therapy efficacy compared to JAK inhibitors. The significant suppression of disease progression observed in mice with RA following treatment with CB provides strong validation for the concept of targeting CXCR4 using CB-like molecules as a potential therapeutic strategy for arthritic conditions. Indeed, CB exhibits all the essential characteristics of a promising new drug for the treatment of rheumatoid arthritis (RA). It is a small molecule that has demonstrated an absence of side effects in *in vivo* preclinical models. CB specifically targets the widely expressed immune cell receptor CXCR4, and it exerts a broad anti-inflammatory effect by modulating cytokine production. These attributes collectively highlight CB as a potential candidate for RA therapy.

In summary, the minimal cytotoxicity of CB, coupled with its wide-ranging inhibitory effects on ex vivo production of inflammatory cytokines and its therapeutic efficacy demonstrated *in vivo*, indicates that the use of CB-like molecules to target CXCR4 could serve as a novel and promising therapeutic strategy for chronic inflammatory diseases, including rheumatoid arthritis.

## Materials and methods

### Blood samples isolation and culture of blood leukocytes

The blood samples obtained from donors in good health were sourced from “Etablissement Français du Sang” (agreement # 07/CABANEL/106), located in Paris, France. The use of materials from JIA patients was conducted with the approval of the Comité de Protection des Personnes (N° EudraCT: 2018-A01358-47) in France. The experimental procedures involving human blood adhered to the guidelines set by the European Union and the Declaration of Helsinki. Informed consent was obtained from all donors, including both healthy individuals and patients. **Table 1** provides a summary of the clinical data pertaining to the JIA patients. *In vitro* experiments were conducted using human mononuclear cells obtained from peripheral blood or synovial fluid (SF) through centrifugation using density gradient medium (STEMCELL Technologies). Human monocytes were isolated by positive selection using Human CD14 microbeads (Miltenyi). SFMC (Synovial fluid mononuclear cells), PBMC (Peripheral blood mononuclear cells), and monocytes were cultured in RPMI 1640 medium (Invitrogen, Gaithersburg, MD) (R10) supplemented with 10% heat-inactivated fetal bovine serum and 1mM glutamine (Hyclone, Logan, UT). The JIA patients were identified as P1 to P36 (refer to **Figure 2**).

**Table 1:** Clinical information of recruited patients.

Patients	Age	Gender	Disease	Treatment	TNF-α (pg.ml) - Blood			TNF-α (pg.ml) - SF		
					plasma	Null	CB	plasma	Null	CB
P1	18	F	oligoJIA	apranax	1,9	–	–	25,6	–	–
P1’	18	F	oligoJIA	apranax	0,7	–	–	22,3	–	–
P2	5	F	oligoJIA	ibuprofen		–	–	26,5	331,1	83,3
P3	5	F	oligoJIA	ibuprofen	18,1	–	–	16,4	2,6	0,5
P4	13	F	oligoJIA	naproxen		–	–	3,5	–	–
P5	10	F	oligoJIA ANA+	voltaren + inexium	2,4	1,7	0,3	11,1	–	–
P6	16	M	ERA	celecoxib	–	–	–	1,6	2,0	0,1
P7	17	M	polyJIA RF-	Tocilizumab	–	13,2	1,1	16,1	19,1	4,1
P8	15	F	polyJIA RF-	tocilizumab + abatacept	–	–	–	13,8	0,6	0,0
P9	5	F	polyJIA	tocilizumab + cortansyl	1,2	3,4	0,2	–	94,8	4,8
P9’	5	F	polyJIA	tocilizumab + cortansyl		1,0	0,0	9,3	–	–
P10	17	M	polyJIA RF-	methotrexate	–	–	–	11,6	2,9	0,1
P11	15	F	oligoJIA	untreated	1,4	4,9	0,1	5,3	2,3	2,6
P12	11	F	oligoJIA	untreated	–	–	–	7,5	18,9	2,0
P13	15	M	oligoJIA	untreated	3,7	0,3	0,3	5,2	1,0	21,6
P14	16	F	oligoJIA	untreated	–	–	–	10,5	57,4	0,1
P15	5	F	oligoJIA ANA+	untreated	–	7,7	14,7	14,4	–	–
P16	17	M	polyJIA RF- ANA+	untreated	2,1	3,4	1,4	3,6	–	–
P16’	17	M	polyJIA RF- ANA+	untreated	–	–	–	7,4	–	–
P17	16	M	ERA	celecoxib	–	–	–	–	1,6	–
P18	11	F	oligoJIA ANA+	methotrexate + adalimumab	–	–	–	–	17,0	0,0
P18’	11	F	oligoJIA ANA+	methotrexate + adalimumab	–	–	–	–	17,9	1,5
P18’’	11	F	oligoJIA ANA+	Methotrexate + Adalimumab	–	–	–	–	25,0	2,4
P19	18	F	oligoJIA	untreated	–	–	–	–	24,0	2,4
P20	16	F	oligoJIA	untreated	–	–	–	–	1,6	0,5
P21	12	F	oligoJIA	untreated	8,8	–	–	–	1,0	34,1
P22	14	M	ERA	Adalimumab	21,8	1,8	0,2	–	–	–
P23	17	F	oligoJIA	Infliximab + Methotrexate + prednisone	23,7	0,3	1,1	–	–	–
P24	13	M	polyJIA	Cortancyl + Arava		7201,1	1620,7	–	–	–
P24’	13	M	polyJIA	Cortancyl + Arava	8,5	1,6	0,0	–	–	–
P25	18	F	sJIA	Ruxolitinib + Prednisone	3,2	0,4	0,3	–	–	–
P26	18	F	oligoJIA	Methotrexate	13,2			–	–	–
P27	18	M	polyJIA RF- ANA+	Methotrexate + Cortancyl	0,0	1,0	0,1	–	–	–
P28	3	F	oligoJIA	untreated	3,3	1,7	0,1	–	–	–
P29	17	F	oligoJIA	untreated	10,5	–	–	–	–	–
P30	13	M	oligoJIA	untreated	4,0	–	–	–	–	–
P31	5	F	oligoJIA ANA+	untreated	3,3	–	–	–	–	–
P31’	5	F	oligoJIA ANA+	untreated	3,5	–	–	–	–	–
P31’’	5	F	oligoJIA ANA+	untreated	3,3	–	–	–	–	–
P32	5	F	sJIA	anti-IL-1	–	2,2	6,3	–	–	–
P33	12	F	sJIA	Tocilizumab + Cortancyl + Methotrexate	–	27,9	980,3	–	–	–
P34	5	F	polyJIA RF-	Tocilizumab	–	13,5	1,1	–	–	–
P35	18	F	sJIA	Tocilizumab	–	0,0	1,3	–		
P36	16	M	sJIA	Tocilizumab + Cortancyl	–	12,1	486,9	–	–	–

SF, synovial fluid; oligoJIA, oligoarticular juvenile idiopathic arthritis; ERA, enthesitis-related arthritis; polyJIA, polyarticular juvenile idiopathic arthritis; sJIA, systemic juvenile idiopathic arthritis; RF, rheumatoid factor; ANA, antinuclear factor. The Null and CB columns correspond to TNF-α assay data by Simoa performed on supernatants of isolated monocytes from blood or synovial fluid of JIA patients. Null: untreated monocytes, CB: monocytes treated with 20 μM CB.

### Cell stimulation

PBMCs were initially seeded at a concentration of 2.106 cells per milliliter (mL), while monocytes isolated from either PBMCs or SFMCs were seeded at a concentration of 1.10^6^ cells per mL. Prior to a 16-hour stimulation with the TLR-7/8 agonist Resiquimod – R848 at a concentration of 5 µg/mL or as specified, the cells were pre-treated for 1 hour with AMD3100 (Sigma-Aldrich) and/or clobenpropit (CB) (Sigma Aldrich) at a concentration of 20 µM (or other specified concentration). Subsequently, flow cytometry or mass cytometry techniques were employed to collect the cells, and supernatants were collected to detect cytokine levels. For intracellular staining, Brefeldin A (BFA) was added to the cells 30 minutes after stimulation and incubated for 5 hours.

THP1-dual cells (Invivogen) were seeded at a concentration of 1.106 cells per mL in RPMI 1640 (Invitrogen, Gaithersburg, MD) (R10) medium supplemented with 10% heat-inactivated fetal bovine serum, 2mM glutamine (Hyclone, Logan, UT), 25mM HEPES, and 1% Pen-Strep. Before a 24-hour stimulation with the TLR-7/8 agonist Resiquimod – R848 at a concentration of 5 µg/mL, the cells were pre-treated for 1 hour with clobenpropit (CB) (Sigma Aldrich) or Histamine at concentrations ranging from 1 to 100 µM. The activity of the NF-κB reporter was measured using QUANTI-Blue, a SEAP detection reagent, following the instructions provided by the supplier.

### Mass cytometry

Peripheral blood mononuclear cells (PBMCs) obtained from healthy donors were stimulated with R848 in the presence of CB. Brefeldin A was added overnight to the culture. Following stimulation, PBMCs were treated with a mixture of surface antibodies and Rh isotopes in PBS for 20 minutes at room temperature. Both commercially available and custom-conjugated antibodies were used to create a panel for phenotypic and functional analysis. Two DNA interchelators, Rhodium isotope mass 103 and Iridium isotopes mass 191 & 193, were employed to determine cell viability and gate the singlet cells during analysis. Prior to analysis, each antibody was titrated using PBMCs. Subsequently, the cells were fixed with Fix-I solution (Maxpar Fix-I buffer; Fluidigm) for 15 minutes at room temperature. After fixation, the cells were incubated with anti-cytokine antibodies and Ir isotopes in Perm-S solution for 30 minutes at room temperature (Maxpar Perm-S buffer; Fluidigm). Before CyTOF acquisition, the cells underwent three washes with highly pure water and were resuspended in water at a maximum concentration of 500,000 cells/mL (Maxpar Water; Fluidigm). Data acquisition was performed using a CyTOF2 instrument (Fluidigm) and the analysis included data cleaning using FlowJo software and the application of the viSNE algorithm in Cytobank for the primary analysis. The table (**Table 2**) provides the details of antibodies coupled with the respective metals.

**Table 2:** Antibodies for mass cytometry.

Targets	Metals	Clones	Suppliers	References
CD45	89Y	HI30	Fluidigm	3089003B
CD38	112Cd	HIT2	Life technologies	Q22150
HLA-DR	115 In	L243	Ozyme	BLE307651
IL-1β	141 Pr	8516	R & D Systems	MAB201-100
CD27	142 Nd	O323	Ozyme	BLE302802
CD123	143 Nd	6H6	Ozyme	BLE306002
CD4	145 Nd	RPA-T4	Ozyme	BLE300516
CD8	146 Nd	RPT-T8	Ozyme	BLE301018
CD86	148 Nd	FM95	Miltenyi	130-095-212
IL-17	151 Eu	CZ8-23G1	Miltenyi	130-095-212
CD304 (BDCA4)	152 Sm	AD5-17F6	Miltenyi	130-095-212
CD11C	153 Eu	Bu15	Ozyme	BLE337202
Il-6	154 Sm	MG2-13A5	Miltenyi	130-095-212
CD279 (PD-1)	156 Gd	PD1.3.1.3	Miltenyi	130-095-212
CD14	160 Gd	M5E2	Ozyme	BLE301810
IFNγ	161 Dy	45-15	Miltenyi	130-095-212
CD185 (CXCR5)	162 Dy	REA103	Miltenyi	130-095-212
CCR7 (CD197)	163 Dy	REA108	Miltenyi	130-095-212
IFN α2	165 Ho	3C5	R & D Systems	H00003440-M27
TNF-α	166 Er	cA2	Miltenyi	130-095-212
CD56	168 Er	HCD56	Ozyme	BLE318324
CD45RA	169 Tm	T6D11	Miltenyi	130-095-212
CD3	170 Er	BW264/56	Miltenyi	130-095-212
IgM	172 Yb	PJ2-22H3	Miltenyi	130-095-212
IgD	174 Yb	IgD26	Miltenyi	130-095-212
CXCL10	175 Lu	REA334	Miltenyi	130-095-212
CD19	176 Yb	LT19	Miltenyi	130-108-029
CD278 (ICOS)	198 Pt	REA183	Miltenyi	130-095-212
CD16	209 Bi	3G8	Fluidigm	3209002B

### Flow cytometry

The cells underwent a PBS wash and were subsequently treated with a viability stain, Zombie Aqua (Biolegend), for 30 minutes at a temperature of 4°C. Following the wash, the cells were suspended in PBS containing 2% FCS and 2mM EDTA and stained with the extracellular mix, APC Vio770 anti CD14 (clone REA599) from Miltenyi Biotec, at a dilution of 1/100. For intracellular staining of TNF-α and IL-1β, an Inside Stain kit (Miltenyi Biotec) was utilized in accordance with the manufacturer’s instructions. In brief, the cells were fixed with 250 µL of the Inside Fix solution for 20 minutes at room temperature (RT), then washed and stained with 100 µL of the Inside Perm solution containing PE anti TNF-α (clone cA2, Miltenyi Biotec) and APC anti-IL-1β (clone REA1172, Miltenyi Biotec) antibodies at a dilution of 1/50 for 30 minutes at RT. Data acquisition was carried out using a Canto II flow cytometer and analyzed using Diva software (BD Biosciences, San Jose, CA) and FlowJo software (Treestar, Ashland, OR).

### CXCR4 knockout experiments

The experiment involved seeding monocytes at a density of 105 cells/100 μl in 96-well plates and placing them in an incubator at 37°C. Two types of siRNA, namely control siRNA (qiagen) and CXCR4 siRNA (SMARTPool, Dharmarcon), were diluted in DOTAP (1,2-dioleoyl-3-trimethylammonium-propane; Roche Applied Sciences). The mixture was gently combined and allowed to incubate at room temperature for 15 minutes. Following the incubation period, the mixture was added to the cultured cells, achieving a final concentration of 160 nM. The cells were then further incubated at 37°C for 24 hours prior to the addition of treatments and stimulation.

### Cytokine detection

The supernatants underwent cytokine production testing through two different methods. The first method involved utilizing the LEGENDplex Antivirus Human panel bead assay (Biolegend, San Diego, USA) as per the manufacturer’s instructions. Alternatively, a commercial Luminex multi-analyte assay (Biotechne, R&D systems) was also employed, following the provided instructions.

### Simoa

A novel Simoa digital ELISA specific to TNF-α was developed by employing a Quanterix Homebrew Assay and incorporating two commercially available antibodies (28). Initially, the 28401 antibody (R&D) clone was utilized as a capture antibody following the coating of paramagnetic beads (0.3 mg/mL). The ab9635 polyclonal antibody (Abcam) was biotinylated (biotin/Ab ratio = 30:1) and employed as the detector. To establish the standard curve, recombinant TNF-α (R&D) was used after conducting cross-reactivity assessments. The limit of detection was determined by calculating the average value of all blank runs ± 3 standard deviations (SDs), resulting in a detection limit of 1 fg/mL.

### RNA isolation and real-time quantitative RT-PCR analyses

The THP1-Dual monocyte cells were cultured at a density of 1.6.106 cells/mL. Prior to a 24-hour stimulation with R848 at a concentration of 10 µg/mL, the cells were pre-treated with clobenpropit (CB) for 1 hour. To isolate the total RNA, an E.Z.N.A. kit (Omega Bio-Tek, USA) was used following the manufacturer’s instructions. For the synthesis of first-strand cDNA, the Prime Script RT Master Mix kit (Takara Bio Europe, France) was employed. Quantitative real-time PCR was conducted at 60°C using the Takyon ROX SYBR MasterMix (Eurogentec, Belgium) in the CFX384 Touch Real-Time PCR Detection System (Bio-Rad, France). The RT-qPCR analyses utilized the primers provided in the list below:

*RPL13A*: forward primer, 5’-AACAGCTCATGAGGCTACGG-3’; reverse primer, 5’-TGGGTCTTGAGGACCTCTGT-3’

*IL1B*: forward primer, 5’-CCTGTCCTGCGTGTTGAAAGA-3’; reverse primer, 5’-GGGAACTGGGCAGACTCAAA-3’

*IL6*: forward primer, 5’-GACAGCCACTCACCTCTTCA-3’; reverse primer, 5’-CCTCTTTGCTGCTTTCACAC-3’

*TNF-α:* forward primer, 5’-CCTGCTGCACTTTGGAGTGA-3’; reverse primer, 5’-GAGGGTTTGCTACAACATGGG-3’

### Nanostring gene expression analysis

Isolated monocytes from both control subjects and patients were used to extract total RNA. The extracted RNA was diluted to a concentration of 20 ng/µl using ribonuclease-free water. Subsequently, 100 ng (5 µl) of each sample was subjected to analysis utilizing the Human Immunology kit v2 and Nanostring Counter. To ensure accuracy, each sample underwent individual multiplexed reactions, consisting of eight negative probes and six serial concentrations of positive control probes. The resulting data was then imported into the nSolver analysis software (version 2.5) for quality assessment and data normalization, adhering to NanoString analysis guidelines. This normalization process involved the utilization of positive probes and housekeeping genes. Prior to hierarchical clustering using Qlucore Omics Explorer version 3.1, the mRNA expression levels were logarithmically transformed. For pathway analysis, the DAVID bioinformatics databank (https://david.ncifcrf.gov) was employed. Supplementary data includes the presented mRNA levels.

### Mice

Washington Biotechnology, INC conducted animal experiments in a manner that ensured unbiased results. The animals were housed in a controlled environment with regulated temperature, humidity, and 12-hour light/dark cycles. They had access to food and water without restriction. The mouse experiments conducted by Washington Biotechnology, INC were reviewed and approved by the WBI Animal Care and Use Committee under the reference IACUC NO. 17 006. The experimental procedures followed the guidelines outlined in the “Guide for the Care and Use of Laboratory Animals” and adhered to ethical standards and regulations. On Day 0, the mice were weighed, and the thickness of their hind limbs was measured using a digital caliper. They were then subcutaneously injected with a 50 µL emulsion of collagen and Complete Freund’s Adjuvant at the base of their tails. On Day 21, the mice were weighed again and received a subcutaneous injection of a 50 µL emulsion of collagen and Incomplete Freund’s Adjuvant at the base of their tails. After this, the mice were divided into five groups, each consisting of eight mice. From Day 21 to Day 35, the first group received intraperitoneal injections of PBS. The second group was administered prednisolone (Sigma, P4153) orally via daily gavage at a dosage of 10 mL/kg, which had been dissolved in 3 mL of PBS to form a 1.5 mg/mL solution. The mice in groups 3, 4, and 5 were treated with CB. Briefly, CB was dissolved in PBS and administered daily through intraperitoneal injections at dosages of 3 mg/kg, 10 mg/kg, and 30 mg/kg respectively (groups 3 to 5). On Day 35, all mice were weighed, assessed for signs of arthritis, and their hind paw volumes were recorded. The mice were anesthetized and their blood was collected in pre-chilled EDTA tubes. The blood samples were processed to obtain plasma, which was then frozen and subsequently thawed at room temperature. The plasma samples were diluted 1:2 and analyzed using ELISA to measure levels of IL-1β (R&D Systems, Cat. MLB00C) and IL-6 (R&D Systems, Cat. M6000B). The limbs were individually removed and preserved in 10% neutral buffered formalin.

### Histology on mice limbs

Formalin-fixed mouse paws were processed routinely, sectioned at approximately 8 microns, and stained with hematoxylin and eosin. Glass slides were evaluated using light microscopy by a board-certified veterinary pathologist. The severity of histologic findings was scored using the following scoring criteria as adapted from Crissman et al (29).

• Inflammation

0=Normal

1=Minimal infiltration of inflammatory cells in synovium and periarticular tissue of affected joints

2=Mild infiltration, if paws, restricted to affected joints

3=Moderate infiltration with moderate edema, if paws, restricted to affected joints

4=Marked infiltration affecting most areas with marked edema

5=Severe diffuse infiltration with severe edema

• Cartilage Damage

0=Normal

1=Minimal=minimal to mild loss of toluidine blue staining with no obvious chondrocyte loss or collagen disruption in affected joints

2=Mild=mild loss of toluidine blue staining with focal mild (superficial) chondrocyte loss and/or collagen disruption in affected joints

3=Moderate=moderate loss of toluidine blue staining with multifocal moderate (depth to middle zone) chondrocyte loss and/or collagen disruption in affected joints

4=Marked=marked loss of toluidine blue staining with multifocal marked (depth to deep zone) chondrocyte loss and/or collagen disruption in most joints

5=Severe =severe diffuse loss of toluidine blue staining with multifocal severe (depth to tide mark) chondrocyte loss and/or collagen disruption in all joints

• Bone Resorption

0=Normal

1=Minimal=small areas of resorption, not readily apparent on low magnification, rare osteoclasts in affected joints

2=Mild=more numerous areas of, not readily apparent on low magnification, osteoclasts more numerous in affected joints

3=Moderate=obvious resorption of medullary trabecular and cortical bone without full thickness defects in cortex, loss of some medullary trabeculae, lesion apparent on low magnification, osteoclasts more numerous in affected joints

4=Marked=Full thickness defects in cortical bone, often with distortion of profile of remaining cortical surface, marked loss of medullary bone, numerous osteoclasts, affects most joints

5=Severe=Full thickness defects in cortical bone and destruction of joint architecture of all joints

### Statistics

Cellular data sets were analyzed by Mann-Whitney test to compare the group with CB treatment to the untreated group. Flow cytometry data were performed using FlowJo software.

Animal data are shown as median and min/max. Data sets were analyzed by ANOVA (Kruskal-Wallis) tests with Dunn’s post-test for multiple comparisons with the different concentrations of CB and prednisolone treatment. Data analysis and graph preparation were performed using GraphPad Prism 8 software (GraphPad Software, San Diego, CA). Statistical significance was determined with a threshold of p < 0.05, indicating values below this threshold were considered statistically significant. Heatmaps were generated using Qlucore OMICS explore Version 3.5 (26) software.

## Data availability statement

The data presented in the study are deposited in the Gene Expression Omnibus repository, accession number GSE243512.

## Ethics statement

The blood from healthy donors was obtained from “Etablissement Français du Sang” (convention # 07/CABANEL/106), Paris, France. For JIA patients’ material, the study was approved by the Comité de Protection des Personnes (N° EudraCT: 2018-A01358-47) in France. Written informed consent to participate in this study was provided by the participants’ legal guardian/next of kin.

## Author contributions

NB, NS, MR and J-PH conceived and designed the study. NB, NS, and J-PH had complete access to all the data generated in the study and assume full responsibility for the data’s integrity and the accuracy of the data analysis. NB, NS, PQ, and MR contributed to writing the paper. BS and J-PH wrote the paper. NB, NS, BB, TD, AB and J-PH performed data analysis. RM, SB, BBM, BT and PQ were involved in the clinical study and sample collection. BB, AL, TD, AB, SN, and MR performed specific experiments and/or analysis. All authors participated in the review process of the manuscript and provided their final approval for the version that is intended for publication. All authors unanimously take responsibility for all aspects of the work, ensuring that any questions concerning the accuracy or integrity of any part of the research are thoroughly investigated and appropriately resolved.
