# Supplementary material for: Targeting the chemokine receptor CXCR4 with histamine analog to reduce inflammation in juvenile arthritis
Source: Front Immunol. 2023 Sep 26;14:1178172. doi: 10.3389/fimmu.2023.1178172 (PMC10562697; doi:10.3389/fimmu.2023.1178172)
Supplement: Supplementary file 1 [file DataSheet_1.docx]

Supplementary data: table showing Nanostring mRNA levels in monocytes from blood from three healthy donors (HD) and three oligoJIA patients.
